# Reflecting on the symmetry of ecosystem tipping points: The influence of trait dissimilarity and environmental driver dynamics in a simple ecosystem model

**DOI:** 10.1002/ece3.11421

**Published:** 2024-08-07

**Authors:** Pascal Bärtschi, Owen L. Petchey

**Affiliations:** ^1^ University of Zurich Zurich Switzerland

**Keywords:** degradation, ecosystem model, environmental change, hysteresis, recovery, symmetry

## Abstract

Our understanding of the similarity in trajectories of ecosystem changes during different directions of environmental change is limited. For example, do the dominant organisms exhibit the same responses to different directions of environmental change, that is, do they exhibit symmetric responses? Here, we explore whether such *response symmetry* is determined and controlled by the symmetry in the features of the underlying biological system (i.e., *system symmetry*), such as in the network and strength of biotic and abiotic processes, and in symmetry of the environmental change (i.e., *environmental symmetry*). For this exploration, we developed and used a simple mathematical model of a microbial ecosystem driven by mutual inhibition in which we could vary the amount of system and environmental symmetry. Our results show that perfect system and environmental symmetry indeed produce perfect response symmetry. Moreover, introducing asymmetry in biological systems or in the environment proportionally increases response asymmetry. These findings suggest using symmetries in ecosystem structure and interaction strength to better understand and predict similarities in degradation and restoration phases of environmental change.

## Graphical abstract

Our understanding of the similarity in trajectories of ecosystem changes during different directions of environmental change is limited. This research article suggests using symmetries in ecosystem structures and interaction strength to better understand and predict similarities in degradation and restoration phases of environmental change.

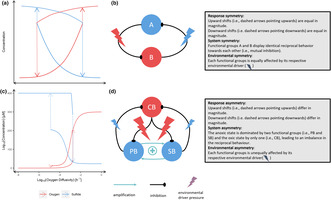

## 1 INTRODUCTION

Changing environmental conditions like temperature, nutrient, and toxin concentrations can cause ecosystems to change (Holling, 1973; Tilman & Lehman, 2001). Depending on many features of the ecosystem, including interactions between and within the biotic and abiotic components (Jorgensen, 2009), such changes can occur in various patterns (Walther, 2010). One type of response is when a small change in an environmental condition causes a large and rapid change in the state of the ecosystem. Examples of such responses include collapse of fishery stocks (Jones & Walters, 2011; Peterman, 1977; Walters & Kitchell, 2001), invasions of exotic species (Mack et al., 2000; With et al., 2002) and changes in aquatic (Scheffer et al., 1993) and terrestrial (Dublin et al., 1990) vegetation.

Such abrupt and large responses, sometimes termed catastrophic shifts (Scheffer et al., 2001) are of considerable importance for ecosystem conservation and management (Beisner et al., 2003; Bush et al., 2017; Da Silveira Lobo Sternberg, 2001; Scheffer et al., 2001; Zhang et al., 2003). They can also be associated with hysteresis, whereby reversal of the catastrophic shift occurs only after a large reversal in environmental conditions past the point where the original catastrophic shift occurred (Scheffer et al., 2001). This hysteresis phenomenon has important implications for restoration practices, as well as for the prevention of the original shift. Such hysteresis loops are displayed in a wide range of ecosystems in terrestrial, marine, and freshwater habitats (Scheffer et al., 2001).

Catastrophic shifts and hysteresis during environmental change imply a phase of environmental degradation and associated catastrophic shift, and then a phase of environmental recovery with a catastrophic shift back to original state. Ecosystem changes during these two phases are addressed by the recently proposed asymmetric response concept (ARC) (Vos et al., 2023). It has two components: (1) the amount of response symmetry between phases characterized by different directions of environmental change, and (2) the mechanisms responsible for response symmetry (or lack thereof) in these two phases. The ARC also focuses on considering species‐specific tolerance to stressor(s) and biotic interaction patterns to understand (a)symmetry in ecosystem response (i.e., system and environmental symmetry).

**FIGURE 1:**
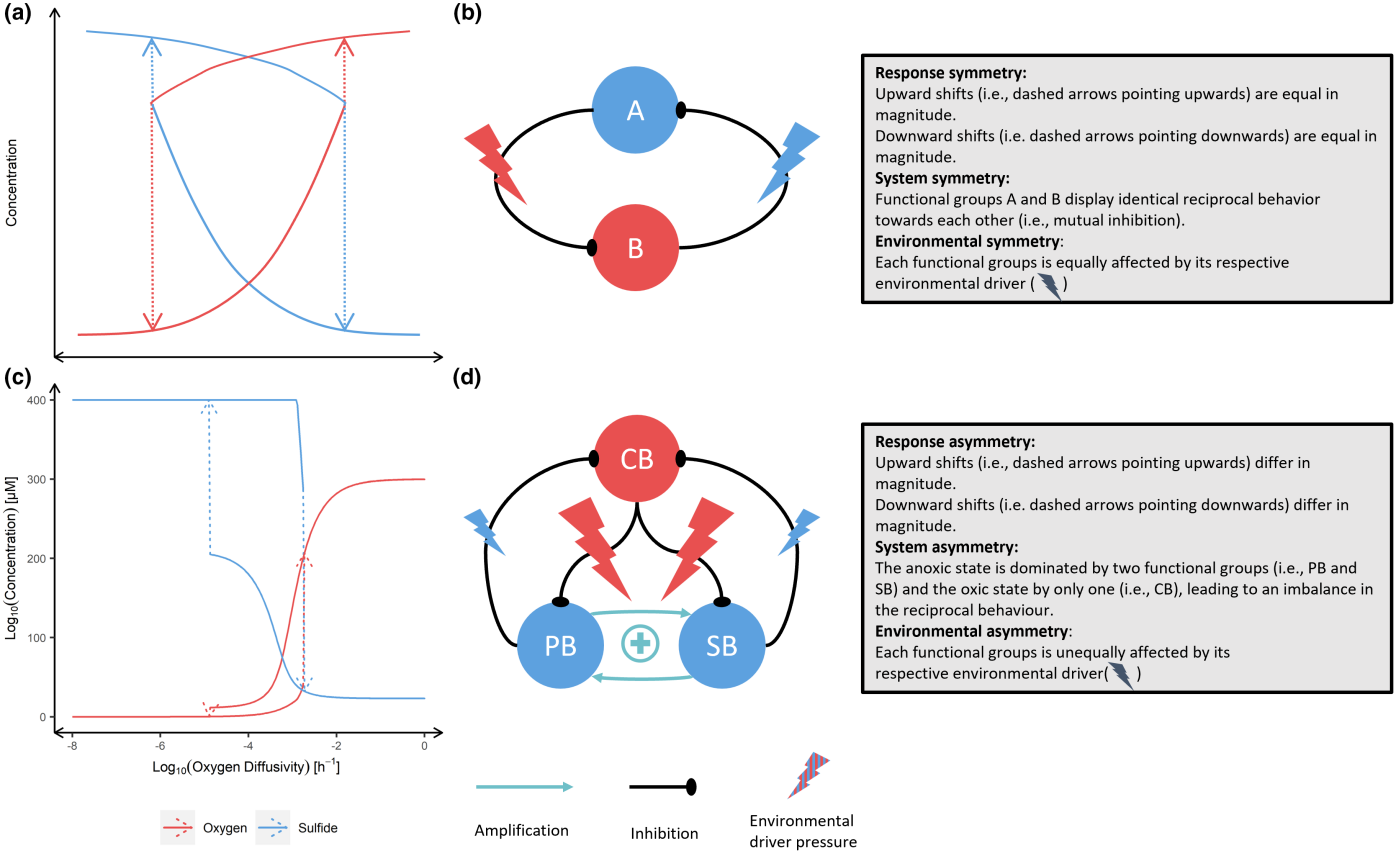
Comparison of long‐term behavior between response symmetry (a) and response asymmetry (c) (Bush et al., 2017). The direction and magnitude of upward and downward shifts of ecosystem state variables (i.e., oxygen in red and sulfide in blue) are depicted with dashed arrows. (a) shows a sketch of the trajectories of response symmetry and (b) visualizes system and environmental symmetry as explained in the boxes. (c) shows a reproduced simulation of Bush et al. (2017) that displays response asymmetry and (d) visualizes system and environmental asymmetry exhibited by the microorganism system found by Bush et al. (2017).

System symmetry (Figure 1b) and asymmetry (Figure 1d) refer to similarity in the structure and parameters of the system. For example, a system with equal numbers of species in the two functional groups that dominate each of two alternate states would be more symmetric than one with different numbers of species in the two functional groups. Environmental symmetry (Figure 1b) and asymmetry (Figure 1d) refer to the environmental drivers that influence the two states of the system. When a driver that favors one state changes greatly, and a driver that favors another state changes little, we say there is asymmetric environmental change.

Furthermore, we define response symmetry as the similarity of the ecosystem response to opposite directions of environmental change. Specifically, we assess response symmetry as the difference in magnitude of the abrupt shift during degradation or recovery of opposite directions of environmental change. If the magnitude of the abrupt shift is equal in both directions of environmental change the symmetry is high (Figure 1a). Low symmetry occurs when the magnitude of the shift differs for recovery or degradation to opposite directions of environmental change (Figure 1c). For instance, there is high response symmetry when the *upward shifts* (i.e., recovery trajectories) of two functional groups are equal in magnitude (Figure 1a). Existing models often display response asymmetry in magnitudes of the upward shifts and also asymmetry in the downward shifts (Bush et al., 2017; Zhang et al., 2003) (Figure 1c) and some empirical data also appear to display such asymmetry (Levy et al., 2004).

Motivated by the asymmetric response hypothesis (ACR) by Vos et al. (2023) this research concerns the symmetry of ecosystem responses to opposite directions of environmental change. It is motivated by the idea to consider (a)symmetries in ecosystems response patterns in both directions of environmental change and the importance of finding responsible mechanisms (i.e., system and/or environmental symmetry) to predict alternative response trajectories and restore ecosystems. However, the hysteresis response pattern is, as described by the “broken leg” model (Sarr, 2002) used by the ACR, classified as *asymmetric*. This is because ecosystems following hysteresis loops do not recover from the same point they degraded. Nevertheless, one could observe response trajectories to opposite directions of environmental change to be similar or dissimilar. For example, for opposite directions of environmental change, functional groups dominating different alternative state may recover or degrade with congruent response trajectories. Relatively little is known about what determines this aspect of ecosystem response symmetry.

This is an important gap in knowledge because ecosystem restoration after environmental degradation is a critical activity. Knowing if one can expect a similar trajectory of change in the recovery phase compared to the degradation phase will be useful for planning purposes. We caution, however, that we use a relatively abstract mathematical model in our research, and direct application would require, for example, development of a case‐specific ecosystem model.

Nevertheless, we are unaware of any study that shows how the amount of response symmetry can be determined and controlled by model and environmental driver characteristics. We address this gap by exploring the response of a simple model ecosystem to two gradually changing stressors (oxygen and sulfide diffusivities). As a foundation for the explored model ecosystem, we take mutual inhibition feedback dynamics between two microbial functional groups (cyanobacteria and sulfur‐reducing bacteria) that can cause hysteresis between oxic and anoxic states of the environment. Such a response is displayed by seasonally stratified, enclosed waters, and is supported by data from marine and freshwater ecosystems experiencing seasons (Martínez‐Alonso et al., 2008; Pjevac et al., 2015; Steenbergen & Korthals, 1982) and can be explained by a mathematical model (Bush et al., 2017). Our mathematical ecosystem model can be driven by two environmental drivers, namely oxygen and sulfide gradients observed in the vertical water column (Martínez‐Alonso et al., 2008). Furthermore, the models' system and environmental symmetry are tuneable, that is, value of traits of the biological and abiotic processes (system symmetry) and the structure of environmental change (environmental symmetry) can be modified to assess their influence on response trajectories (response (a)symmetry).

We predict that when system and environmental symmetry areexhibited, the ecosystem will display perfect response symmetry. It follows that we also expect that greater system or environmental asymmetry results in greater response asymmetry. Moreover, we suggest that our system's symmetry in biotic and abiotic processes and environmental change may be useful to gain further insight into the influence of trait dissimilarities (system asymmetry) or stressor imbalances (environmental asymmetry) on ecosystem response trajectories.

## 2 MATERIALS AND METHODS

### 2.1 Symmetric ecosystem model

Our approach was to modify an existing ecosystem model to make it symmetric in structure and strength of biotic and abiotic processes. The existing model was that of Bush et al. (2017) of oxic‐anoxic regime shifts caused by mutual inhibition between cyanobacteria and sulfur bacteria. To develop this into a symmetric model, we had to reduce the biological realism, for example, by removing one of the three functional groups of organisms so that only two remained. As well as the desirable consequences (i.e., creating a symmetric system) this reduction in biological realism may have undesirable consequences (e.g., less relevance to any specific real ecosystem). We discuss some of the possible undesirable consequences in the Section 4.

**FIGURE 2:**
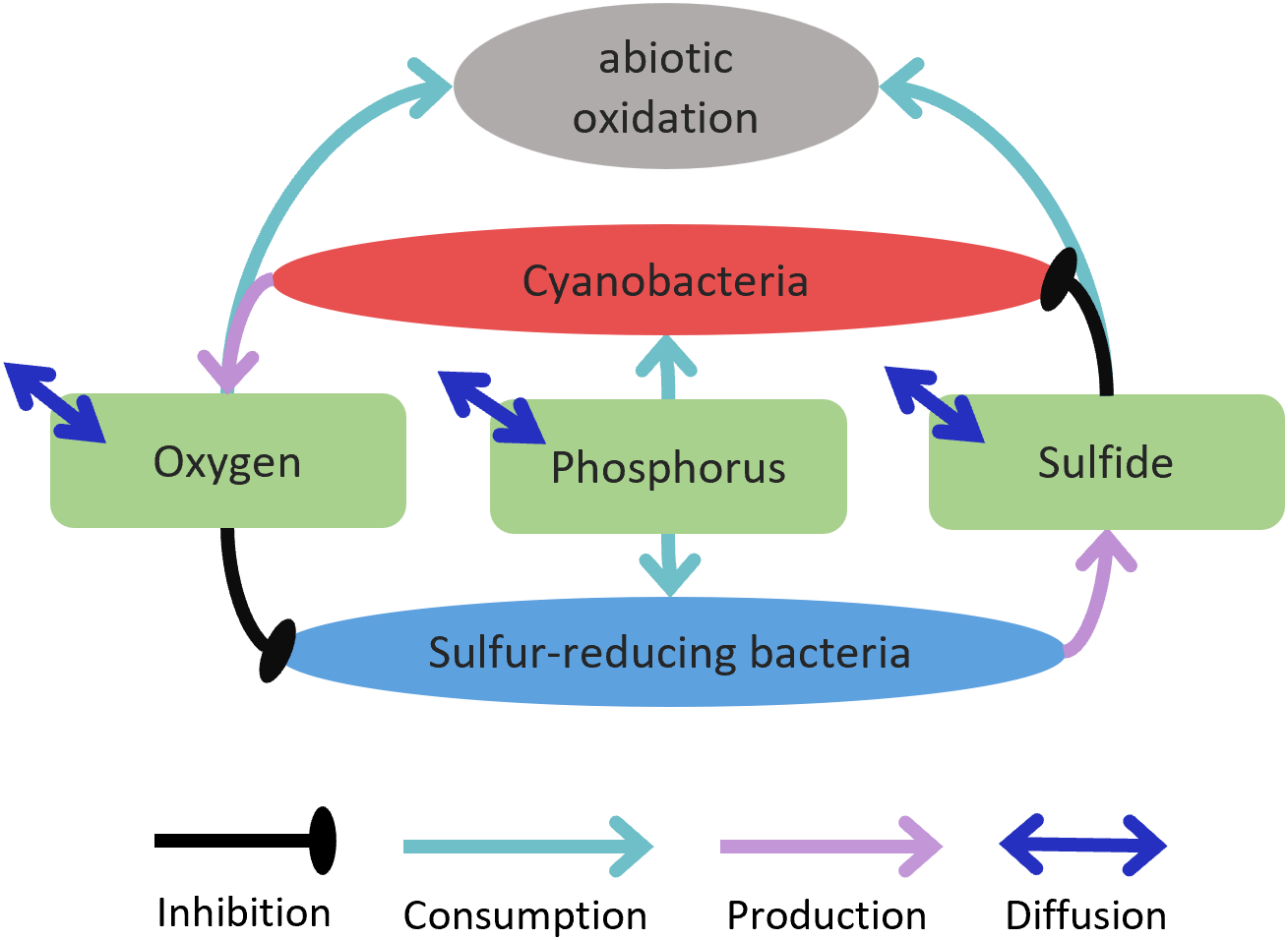
Schematic model diagram of the symmetric model system (system symmetry). Two substrates (i.e., oxygen and sulfide) are produced, one by each of two functional groups (i.e., cyano‐ and sulfur‐reducing bacteria) which hence inhibit the other functional group. Every line is represented in the Equations (1.1), (1.2), (2.1), (3.1), (3.2), (4.1), (4.2), (4.3). Dark blue, bidirectional arrows indicate diffusion of substrate in and out of the system. One‐directional arrows either denote consumption (turquoise) of nutrient (i.e., phosphorus) or production of substrate. Black lines describe growth inhibition.

The modified model is shown in Figure 2. The two functional groups are cyanobacteria and sulfur‐reducing bacteria. Both consume phosphorous and thereby grow and compete. They are each inhibited by a substrate (sulfide and oxygen) that is produced by the other functional group. The two substrates can diffuse in and out of the system, and both react with each other and then are lost from the system. Thus, the system is symmetric in the structure of the processes that affect the abiotic and biotic compartments, that is, it exhibits system symmetry. These modifications were made in the ordinary differential equations used to represent the system. Moreover, the system was developed to be influenced by two environmental stressors, oxygen ($\alpha_{O}$) and sulfide ($\alpha_{S}$) diffusivity.

The interactions depicted in Figure 2 were modeled with a set of differential equations. The rate of change in density of each functional group was growth rate minus death rate:

(1.1) $$\frac{{\mathrm{dN}}_{\mathrm{CB}}}{\mathrm{dt}}=g\left(P\right)h\left(S\right)\;N_{\mathrm{CB}}-mN_{\mathrm{CB}}$$

(1.2) $$\frac{{\mathrm{dN}}_{\mathrm{SB}}}{\mathrm{dt}}=g\left(P\right)h\left(O\right)\;N_{\mathrm{SB}}-mN_{\mathrm{SB}}$$

where $N_{CB}$ is the density of the cyanobacteria and $N_{SB}$ is the density of the sulfur bacteria. The Monod function $g\left(P\right)$ describes the growth rate of a bacterial group in response to the concentration of P (i.e., phosphorus):

(2.1) $$g\left(P\right)=g_{\max}\left(\frac{P}{k_{P}+P}\right)\;$$

where $g_{\max}$ is the maximum specific growth rate and $k_{P}$ the half‐saturation constant in μM. These two parameters were made equal in the two functional groups.

Growth is inhibited according to the Haldane functions $h\left(S\right)$ and $h\left(O\right)$:

(3.1) $$h\left(S\right)=\frac{1}{1+\left(\frac{S}{H_{S}}\right)}$$

(3.2) $$h\left(O\right)=\frac{1}{1+\left(\frac{O}{H_{O}}\right)}$$

where $H_{S}$ and $H_{O}$ are half‐inhibition constants, that is, the concentration of inhibitory substance at which the growth rate is reduced to 50% compared to g(P).

The concentrations of oxygen and sulfide are affected by production (oxygen is produced by the cyanobacteria and sulfide is produced by the sulfur bacteria), by loss due to abiotic oxidation of sulfide, and by diffusion into or out of the system. Accordingly, changes in the concentration of oxygen and sulfide are described by the following two equations:

(4.1) $$\frac{dO}{\mathrm{dt}}=p_{O}g\left(P\right)h\left(S\right)N_{\mathrm{CB}}-c\mathrm{OS}+\alpha_{O}\left(O_{b}-O\right)$$

(4.2) $$\frac{dS}{\mathrm{dt}}=p_{S}g\left(P\right)h\left(O\right)N_{\mathrm{SB}}-c\mathrm{OS}+\alpha_{S}\left(S_{b}-S\right)$$

where O and are the concentrations of oxygen and sulfide, respectively. Furthermore, $p_{O}$ is the production constant of oxygen (μM cell^−1^) by cyanobacteria, $p_{S}$ is the production constant of sulfide (μM cell^−1^) by sulfur bacteria, and c is the oxidation rate. Oxygen diffusion is determined by the difference between the realized oxygen concentration and the background oxygen concentration, multiplied by the oxygen diffusivity parameter $\alpha_{O}$. Likewise, sulfide diffusion is determined by the difference between the realized sulfide concentration and the background sulfide concentration, multiplied by the sulfide diffusivity parameter $\alpha_{S}$.

The concentration of phosphorous is affected by consumption by the two bacterial groups, and by diffusion:

(4.3) $$\frac{dP}{\mathrm{dt}}=-\frac{1}{y_{P}}g\left(P\right)h\left(S\right)N_{\mathrm{CB}}-\frac{1}{y_{P}}g\left(P\right)h\left(O\right)N_{\mathrm{SB}}+\alpha_{P}\left(P_{b}-P\right)$$

where P is the concentration of phosphorus, $y_{P}$ is the yield in cells μM^−1^, *P_b_* is the background concentration of phosphorous, and $\alpha_{P}$ is the phosphorous diffusivity.

These ODEs describe the structure of the system. Symmetry is also a feature of the parameter values, with identical rates of biotic and abiotic processes regarding both functional groups. Literature (Table 1) provides parameters for biotic processes (e.g., growth rate or half‐inhibition constant), whereas abiotic parameters were used to tune the system to display nonlinear hysteresis. System symmetry is exhibited by using the parameter set depicted in Table 1 together with our ODE system, whereas environmental symmetry is created by having both oxygen and sulfide diffusivity changing inversed over the same range at the same time. We refer to this parameter set as a symmetric configuration, while parameters set not displaying either system or environmental symmetry characterize asymmetric configurations.

**TABLE 1:** Parameter values describing a symmetric system configuration.

Parameter	Meaning	Value	Reference
$g_{max}$	Maximum specific growth rate of cyanobacteria and sulfur‐reducing bacteria	0.1 h^−1^	Kalyuzhnyi et al. (1998)
$k_{P}$	Half‐saturation constant of cyanobacteria and sulfur‐reducing bacteria on phosphorus	0.5 μM	Bush et al. (2017)
$H_{S}$	Half‐inhibition constant of sulfide on cyanobacteria	100 μM^−1^	Gerritse et al. (1992)
$H_{S}$	Half‐inhibition constant of oxygen on sulfur‐reducing bacteria	100 μM^−1^	Gerritse et al. (1992)
$y_{P}$	Yield of cyanobacteria and sulfur‐reducing bacteria on phosphorus	1.67 × 10^8^ cells × μM^−1^	Saxton et al. (2012)
$p_{S}$	Production of sulfide by sulfur‐reducing bacteria	3.00 × 10^−8^ μM × cell^−1^	—
$p_{O}$	Production of oxygen by cyanobacteria	3.00 × 10^−8^ μM × cell^−1^	—
m	Mortality rate of cyanobacteria and sulfur‐reducing bacteria	0.04 h^−1^	Bush et al. (2017)
$\alpha_{P}$	Diffusivity of phosphorus	0.1 h^−1^	*Tuning parameter*
$O_{b}$	Background concentration of oxygen	100 μM	*Tuning parameter*
$S_{b}$	Background concentration of sulfide	100 μM	*Tuning parameter*
$P_{b}$	Background concentration of phosphorus	10 μM	*Tuning parameter*
c	Oxidation rate of oxygen and reduced sulfur	0.1 μM × h^−1^	*Tuning parameter*
$\alpha_{O}$	Diffusivity of oxygen	10^−2^ to 10^0^ h^−1^	*Tuning parameter*
$\alpha_{S}$	Diffusivity of sulfide	10^−2^ to 10^0^ h^−1^	*Tuning parameter*

*Note*: For explanation of the origin of the values, see text.

### 2.2 Stable state finding

To investigate response symmetry, following the work of Bush et al. (2017), we investigated how the long‐term final state of the system (assumed to be a stable state) depended on historical conditions, current environmental conditions, and the amount of system and environmental symmetry. This required quantifying the long‐term final state of the system at multiple values of the environmental drivers. We followed the approach of Limberger et al. (2022), which they termed the “temporal approach.” It involves two independent simulations, one in which the environmental driver changes in one direction (e.g., increase) and the other in which it changes in the other direction (e.g., decrease). An important difference in our study was having two varying environmental drivers: oxygen diffusivity and sulfide diffusivity. Hence in one simulation oxygen diffusivity was increased while sulfide diffusivity decreased, and in the other simulation, the pattern was reversed.

More specifically, the increase/decrease was made in a stepwise fashion, with 1,000,000 h (biological time) at each step. This gave sufficient time for the system to stabilize during each step. At the end of each step, the system state was recorded. This gives the system state at each value of the environmental drivers. At the start of a new step, the oxygen and sulfide diffusivity were set to the next values in the sequence, and the system was simulated again for 1,000,000 h (biological time) using the previous final state of the system as the new starting state.

To visualize the temporal dynamics of the system we simulated a diffusivity pattern containing both sulfide diffusivity increasing and decreasing with oxygen diffusivity decreasing and increasing, respectively. Here, also the long‐term final state of the system was considered, but in contrast to the temporal approach, only one diffusivity vector as described above was simulated.

Simulations were initiated with 10^5^ cells L^−1^ per bacteria group, 20 μM per substrate species, and 10 μM of nutrient, that is, phosphorus. To prevent critically low abundances of either bacteria group, causing computational interference, 1 cell L^−1^ was added every 1000 h to both groups. Oxygen and sulfur diffusivities were varied in the range from 10^−2^ to 10^0^ h^−1^.

### 2.3 Asymmetric configurations

To explore response trajectories resulting from asymmetric configurations we changed either the strength of inhibition in one of the two functional groups (i.e., system asymmetry), or we made one of the two environmental drivers change more than the other (i.e., environmental asymmetry). First, the half‐inhibition constant of sulfur‐reducing bacteria was changed sequentially in steps of 10 from 30 up to 170 μM^−1^ (the half‐inhibition constant for the cyanobacteria remained 100 μM^−1^). This way, asymmetries in species‐specific tolerance on response trajectories are investigated.

And second, the extent of sulfide diffusivity change was varied. Recall that in the symmetric case, the diffusivities of both oxygen and sulfur varied from 10^−2^ to 10^0^ h^−1^ (Table 1). To create environmental asymmetry, we increased or decreased the amount of change in sulfur diffusivity by an *environmental asymmetry factor* such that when this factor was less than 1 (e.g., 0.8) then the amount of change was decreased (e.g., 10^−1.8^ to 10^−0.2^). When the factor was greater than 1 (e.g., 1.2) then the amount of change was increased (e.g., 10^−2.2^ to 10^0.2^). In this work, asymmetry factors of 0 (i.e., no change in sulfide diffusivity) to 2 (i.e., 10^−3.0^ to 10^1.0^, meaning greater change compared to oxygen diffusivity) were investigated.

### 2.4 Quantification of (a)symmetry

Assessing the amount of response asymmetry required measuring the magnitude and location of tipping points (Figure 1). The first step is to identify a tipping point. We did this by calculating the change in system state between two consecutive environmental conditions. At a tipping point, this change is large compared to nontipping points, and we used the maximum observed value of this change to identify the location of the tipping point. A tipping point is defined by its location on the oxygen diffusivity gradient and its magnitude (i.e., the extent of change in an ecosystem state across the tipping point).

We assessed the overall response symmetry rather than that of individual state variables to present simpler and clearer results. We assessed overall response symmetry by calculating the total shift magnitude in each direction of environmental change (Figure 5a,c). The total shift magnitude is the sum of the increase and decrease shift magnitudes of the two substrates (oxygen and sulfide) in the same direction of environmental change. For example, the total shift magnitude of the anoxic‐oxic tipping point is the sum of the oxygen increase magnitude and the sulfide decrease magnitude characterizing the regime shift to the oxic state.

### 2.5 Software

All software was written and run in R (R Core Team, 2022). We built our simulation and analysis code on top of the existing *microxanox* R package (Krug & Petchey, 2023). The package was extended with the symmetric model system and functions to assess the (a)symmetry and to visualize properties of response (a)symmetry. Our extension was contributed to the official repository of *microxanox*, leading to the release of package version 0.9.3 that was used for all simulations and analysis in this study. The purpose and usage of our developed software are documented in the symmetry system user guide (https://uzh‐peg.r‐universe.dev/articles/microxanox/SymSys‐User‐guide.html). All simulated data and analysis workflow executed are available in the tipping point github repository (https://github.com/pascalbartschi/tipping‐point‐symmetry).

## 3 RESULTS

**FIGURE 3:**
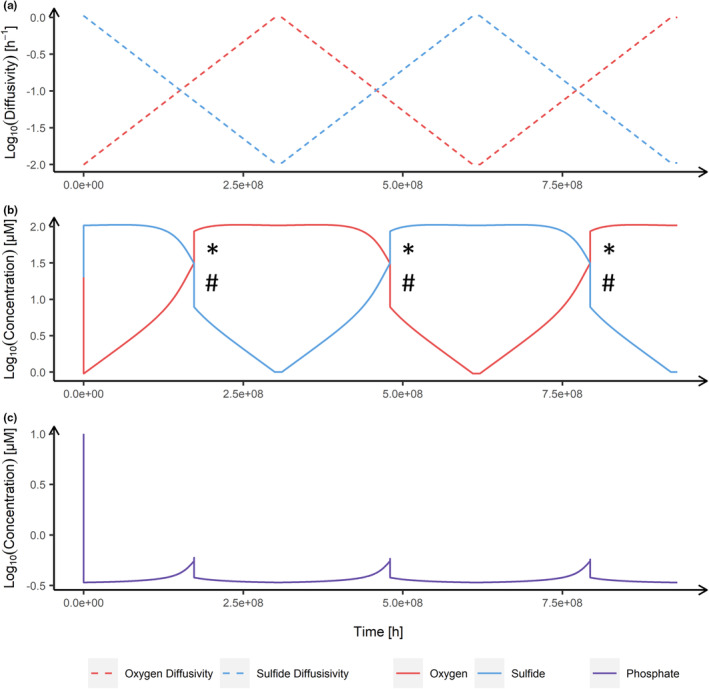
Symmetric temporal response dynamics resulting from system and environmental symmetry. For example, the upward shift magnitudes labeled with asterisks (*) are equal and the three downward shifts labeled with hashes (#) are also equal. (a) Environmental change in sulfide (blue) and oxygen (red) diffusivities. This diffusivity pattern is environmentally symmetric. (b) Trajectories of sulfide (blue) and oxygen (red) concentration over time, responding to environmental change initially favoring the first anoxic state, second the oxic state, and lastly the anoxic state again. (c) Phosphorus concentration (purple) dynamics are independent of the direction of environmental change.

If system and environmental symmetry are present (i.e., identical inhibition parameter values and identical extents of change in the two environmental drivers), the dynamics of the two substrates are identical (Figure 3). Consider when initial environmental conditions favor the anoxic state, due to high sulfur diffusivity (Figure 3a,b). As the sulfur‐reducing bacteria become dominant and rise to 10^9^ cells L^−1^, phosphorus is depleted from 10 to 0.45 μM (Figure 3c). With increasing environmental pressure on the sulfur‐reducing bacteria due to increasing oxygen diffusivity, accompanied with decreasing sulfide diffusivity (Figure 3a), sulfide concentration starts to decrease, along with the increase in oxygen (Figure 3b). Approximately 7.5 × 10^7^ h biological time after oxygen diffusivity becomes higher than sulfur diffusivity, oxygen concentration equalizes with sulfur concentration, which represents the anoxic‐oxic tipping point. At this point, there is a shift from the anoxic state to the oxic state. Furthermore, the shift back to the anoxic state shows identical tipping point characteristics (e.g., magnitude). After the system shifts to the anoxic state, diffusivity of sulfide is decreased (along with increasing oxygen diffusivity) again (Figure 3a), resulting in a second anoxic‐oxic tipping point (Figure 3b). Characteristics regarding dynamics and magnitude of this regime shift are identical to the previous two, including the spikes of phosphorus from 0.45 to 0.55 μM (Figure 3c).

**FIGURE 4:**
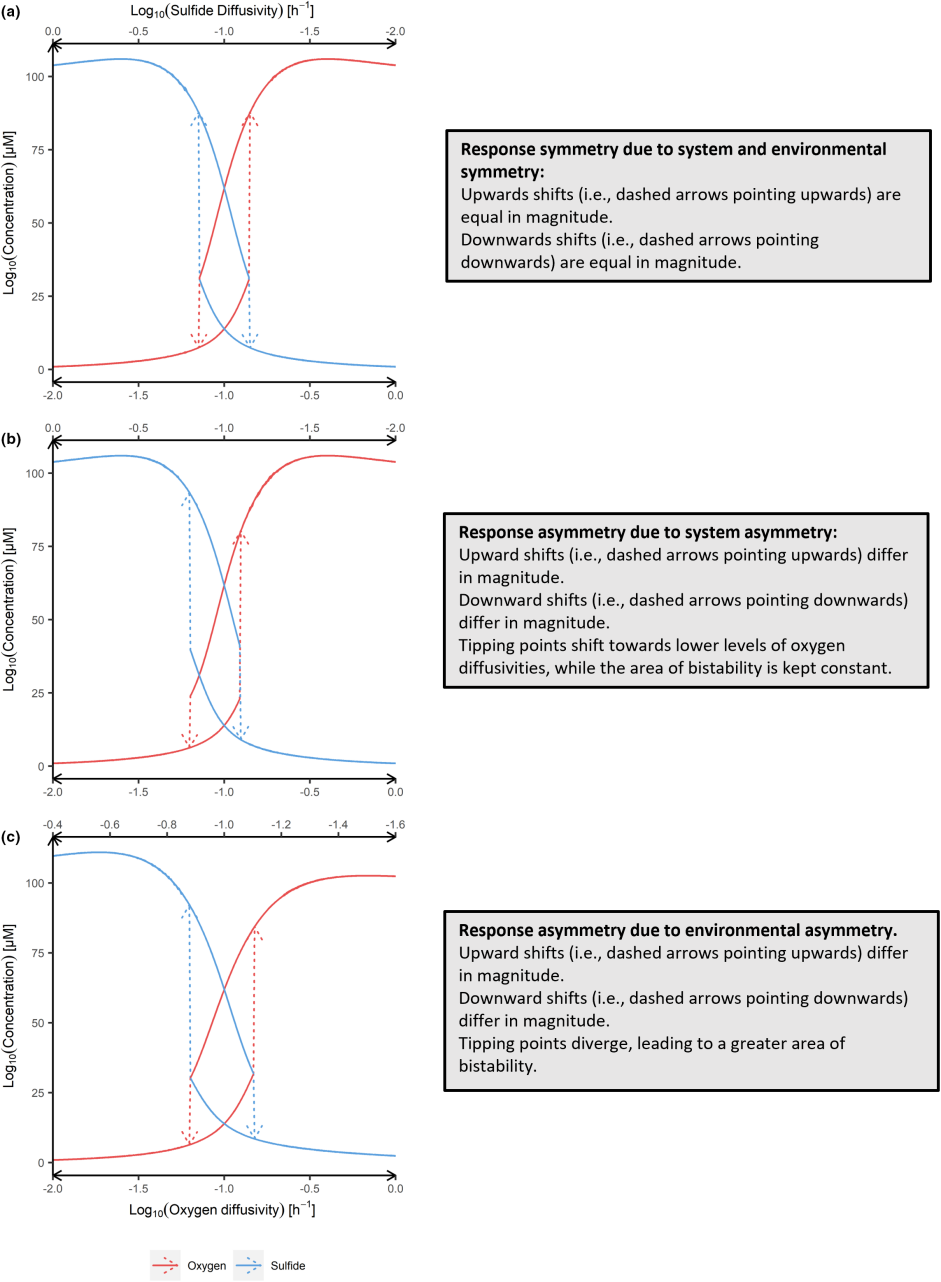
Comparison of the symmetric response (a) with asymmetric response (b,c) resulting from system (b) or environmental asymmetry (c). In (b) system asymmetry was induced by reducing the half‐inhibition constant of sulfur reducing bacteria from 100 to 60 μM and in (c) environmental asymmetry is characterized by 40% less variation of sulfide diffusivity (i.e., asymmetry factor of 0.6).

In summary, we find that the symmetric configuration (Table 1) leads to a symmetric response of the system, as the increase and decrease magnitudes of pairs of corresponding tipping points are identical. That is, in Figure 3b, the three upward shift magnitudes labeled with asterisks (*) are all equal even though two are for oxygen and one for sulfide. The three downward shift magnitudes labeled with hashes (#) are also equal, even though again some are for oxygen and some for sulfide. This symmetry is also depicted in Figure 4a,b.

Examples of response dynamics to symmetric and asymmetric configurations are shown in Figure 4. Here, we induced system (Figure 4b) or environmental (Figure 4c) asymmetry, for example, such that either state is more favored by trait values (e.g., half inhibition constant) or environmental driver pressure, respectively.

System asymmetry results in response asymmetry. For instance, when the half‐inhibition constant of sulfur‐reducing bacteria is decreased by 40% (i.e., from 100 to 60 μM^−1^) both tipping points shift toward lower oxygen diffusivity levels (Figure 4b), while the distance between the tipping points is unchanged compared to the symmetric response (Figure 4a). Furthermore, the oxygen and sulfide downward shift magnitudes (Figure 4b) change in opposite directions compared to the symmetric response (Figure 4a). The oxygen downward shift magnitude doubles, in contrast to the sulfide downward shift magnitude which halves (Figure 4b vs. a).

Environmental asymmetry results in response asymmetry. For example, reducing the amount of change in sulfur diffusivity by log_10_ (0.4) (i.e., stressor asymmetry factor 0.6) causes tipping point locations to diverge unequally. Moreover, the upward shift magnitude (Figure 4e) of oxygen and sulfide changes differently than their downward shift magnitude (Figure 4f). The two downward shift magnitudes remain quite similar (Figure 4f), whereas the upward shift magnitudes increase for oxygen and decrease for sulfide (Figure 4e). In contrast to the symmetric response (Figure 4a,b) or response to system asymmetry (Figure 4c,d), environmental asymmetry (Figure 4e,f) resulted in different substrate equilibria after the increase shift. While in system symmetry and asymmetry cases (Figure 4a–d) both concentrations after the increase are the same at 106 μM, oxygen is dominant in the oxic state at 102 μM and sulfide in the anoxic state at 110 μM (Figure 4e,f).

**FIGURE 5:**
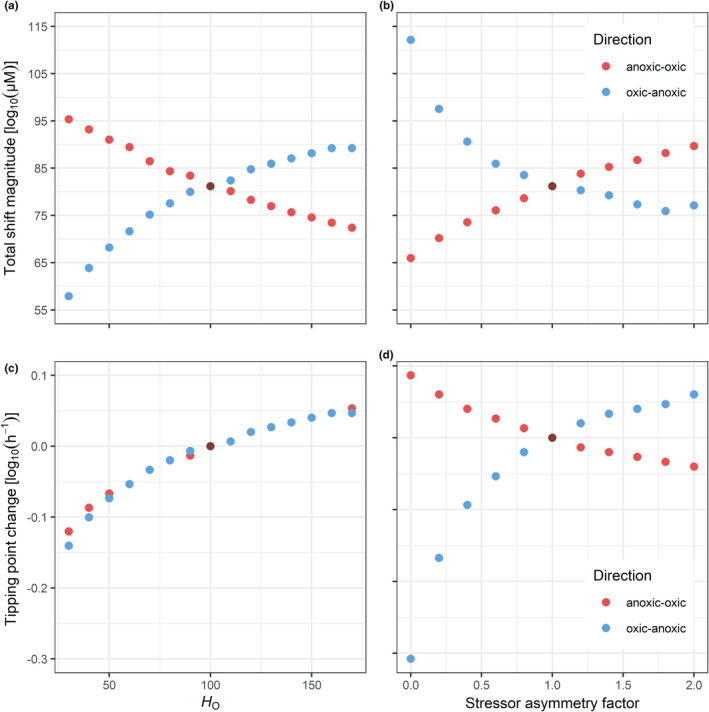
Relationship of system (i.e., the strength of inhibition by oxygen, *H*_o_) and environmental asymmetry with total magnitudes and locations of tipping points. Brown scatters depict the symmetric response. (a, b) show the total oxic‐anoxic and anoxic‐oxic shift magnitude in blue and red, respectively. Similarly, (c, d) show the difference in oxic‐anoxic (blue) and anoxic‐oxic (red) tipping point locations. Positive and negative values mean that tipping points changed to higher values and lower values, respectively. In (a, c) the tolerance of sulfur‐reducing bacteria was manipulated by changing its half‐inhibition constant (i.e., system asymmetry), whereas in (b, d) the pattern of diffusivity change was driven asymmetrically by changing the range of sulfide diffusivities (i.e., environmental asymmetry).

To assess the relationship between increased system or environmental asymmetry to the degree of response asymmetry total shift magnitudes are visualized in Figure 5. First, increasing system asymmetry results in increasing response asymmetry. Setting different values of the half‐inhibition constant of sulfur‐reducing bacteria (*H*_o_), ranging from 30 to 170 μM^−1^, revealed that response symmetry only occurs when the value of this constant was the same for both functional groups (100 μM^−1^) (Figure 5a,b). Increasing *H*_o_ increases the total oxic‐anoxic shift magnitude and decreases the total anoxic‐oxic magnitude. Decreasing *H*_o_ has the opposite effect. Additionally, increasing *H*_o_ results in both tipping points moving to lower levels of oxygen diffusivity (Figure 5c).

And second, also increased environmental asymmetry causes increased response asymmetry (Figure 5b,d). Greater sulfur diffusivity change (asymmetry factor > 1) decreases the total oxic‐anoxic shift magnitude and increases the total anoxic‐oxic magnitude. Lower sulfur diffusivity change (asymmetry factor <1) has the opposite effect (Figure 5b). Increasing environmental asymmetry causes the location of both tipping points to move in opposite directions, either increasing the region of bistability (i.e., the distance between the two tipping points) (asymmetry factor <1) or decreasing it (asymmetry factor > 1) (Figure 5d). In an extreme case, where sulfur diffusivity remains constant and only oxygen diffusivity gradually changes (i.e., asymmetry factor 0, Figure 5b) oxic‐anoxic magnitudes have twice the extent compared to anoxic‐oxic magnitudes. Similarly, the oxic‐anoxic tipping point moves about three times further than the one to the oxic state (Figure 5d).

## 4 DISCUSSION

Developing an ecosystem model that exhibits system and environmental symmetry revealed three key findings. First, system dynamics were identical in the two directions of environmental change (Figures 2 and 3a,b), implying a symmetric response (Figures 3 and 4a,b). That is, a symmetric model can indeed produce symmetric responses while still preserving system bistability, occurrence of tipping points, and hysteresis. Second, the greater the imbalance in the system, that is, the greater the system or environmental asymmetry, the stronger the asymmetric response is exhibited (Figure 5). And third, system and environmental asymmetry had different effects on the character of the response asymmetry, that is, the change in locations of the tipping points. While changing tolerance (i.e., system symmetry) moved both tipping point locations into the same direction, changing the environmental symmetry shifted tipping point locations in opposite directions. This means either reducing (asymmetry factor > 1) or increasing the region of bistability (asymmetry factor <1) (Figure 5d).

The first key finding serves as the foundation of our work, as response symmetry is necessary to relate to asymmetric responses of the system. Assessments of the asymmetric responses are made relative to the symmetric response and only make sense in its context, highlighting its importance. The core of this foundation is that system (Figure 2, Table 1) and environmental symmetry (Figure 3a) produce response symmetry (Figures 3b and 4a,b), while still exhibiting hysteresis.

The second key finding includes that the model exhibits reliable and biologically sound responses when asymmetry is introduced. Greater asymmetries in configurations lead to responses with greater asymmetry (Figure 5). Increased system (Figure 5a,c) and environmental (Figure 5b,d) asymmetry induce higher asymmetry between upward/downward shift magnitudes of oxygen and sulfide. Moreover, the greater system or environmental asymmetries become, the further tipping points move away from their location in the symmetric response.

The third key finding connects the first and the second to explain the character of changing dynamics with higher system asymmetry. Increasing imbalance of tolerance and environmental pressure patterns both show characteristic and biologically sensible effects on the tipping point location. First, increasing the tolerance of sulfur‐reducing bacteria to oxygen leads to collectively moving tipping point locations to higher oxygen diffusivity levels, implying greater stability of the anoxic state (Figure 5c).

Second, the change in the extent of the region of bistability can be explained by changes in environmental asymmetry (Figure 5d). Depending on whether the range of sulfide diffusivity change is smaller or greater, the two thresholds (i.e., the combination of sulfide and oxygen diffusivity characterizing a tipping point) move away from or toward each other. For instance, less variation in sulfide diffusivity leads to the threshold combination defining the anoxic and oxic tipping to occur at lower and higher oxygen diffusivities, respectively. As these thresholds diverge in the diffusivity pattern, the tipping points follow in the ecosystem response (i.e., broader bistability). Similarly, higher variation in sulfide diffusivity causes these thresholds to converge, resulting in a smaller bistability area, as tipping point locations converge.

In summary, for variation in the tipping point location, we found a sensible biological explanation (Figure 5b,d). However, asymmetries in increase, decrease, or total magnitudes are not straightforward to interpret. Increasing tolerance and an increasing stressor asymmetry factor favors sulfur‐reducing bacteria (Figure 5b,d) according to the movements tipping point locations, but the magnitudes of these manipulations show a conflicting pattern (Figure 5a,c). Thus, the magnitudes should primarily be used to assess the amount of symmetry.

According to the ARC by Vos et al. (2023), considering (a)symmetries of patterns of response trajectories in both directions of environmental change and finding responsible mechanisms is important to predict alternative ecological trajectories and restore ecosystems. Our symmetric ecosystem model manifests a first step toward grasping the potential of using symmetry for restoration. It confirms that response asymmetries in hysteresis loops can originate from system and environmental asymmetries.

It is important to note that the (a)symmetry relationships found in this study are a result of the mutual inhibition present in the system. That is, without a mutually reciprocal relationship of some kind, it is likely that there can be no symmetry. Mathematical models show that alternative stable states are possible when positive feedback is strong enough (Holling, 1973; May, 1977; Noy‐Meir, 1975). In system dynamics described by positive feedback dynamics other than mutual inhibition, we cannot be certain that our results would apply. For example, if system dynamics are only described by one single state driven by one environmental variable (Rahmstorf, 1995; Reid et al., 1998) the assessment of asymmetry presented in this research is difficult to apply (i.e., how could the system be made symmetric (Figure 2)). Furthermore, in our study, we did not examine indications of symmetries between upward and downward shifts within one state variable. For instance, in Figure 3, symmetry between upward and downward shifts describes a situation where magnitudes of asterisks (*) and hashtags (#) are equal. However, we suggest that response asymmetry can exclusively be assessed between opposing state variables, which is not the case for systems driven by single variables. It is possible that such systems are themselves simplifications of a more complex system in which there are multiple and opposing state variables, in which symmetry could be examined. Put another way, we simplified the model of Bush et al. (2017) to investigate symmetry, whereas other models of systems would need to be made more complex to allow symmetry to be investigated.

From a management perspective, this model suggests that information about restoration of a collapsed and favored alternative states can be acquired by observing the previous development of the unfavoured state. By accounting for system and environmental asymmetries, observations of developing unfavoured states may be used as the basis of predictions about the development of a favored state during restoration. Similarly, data about the loss of a favored state may be used to find approaches to destabilize an unfavoured state.

In practice, such insights will still require the work of model development. Moreover, the model would need to clearly represent mutual inhibition (Figure 2) to ensure system and environmental symmetry between antagonistic functional groups. In the well‐studied example of shallow lakes, human‐induced eutrophication causes sudden loss of transparency and macrophytes (Jeppesen et al., 1999; Scheffer et al., 1993). This system can be simplified to mutual inhibition between the clear water state dominated by submerged vegetation and the turbid state dominated by phytoplankton. The clear water self‐stabilizes by stronger growth of plants that are exposed to more light and that host phytoplankton grazing *Daphnia*. The turbid state absorbs light, preventing vegetation growth, and favors *Daphnia* consuming fish (Meijer et al., 1999). As in the example of oxic‐anoxic regime shifts (Bush et al., 2017) oxygen and sulfide diffusivity, we can also identify the opposite driver to nutrients being CO_2_ concentration, which decreases with increasing nutrient concentration (Scheffer et al., 1993).

Another example of ecosystems driven by two opposite drivers is coral reefs, which can either be dominated by corals that can host huge biodiversity or alternatively by fleshy, thick macroalgal cover (Knowlton, 1992). Corals self‐reinforce by providing niches for fish and sea urchins which graze early‐stage algae. However, increasing land‐use intensity (i.e., increasing nutrient concentrations) combined with intensive fishing can lead to algae overgrowing corals due to the release of grazers (Scheffer et al., 2001). The coral state is then difficult to recover, as adult macroalgae are palatable and algal cover inhibits settlement of coral larvae (Nyström et al., 2000). Moreover, dynamics of mutual inhibition can be identified in woodlands, where the landscape is either predominantly covered with grass scattered with light wood or alternatively by heavier wood (Dublin et al., 1990). The open grassy landscape is kept open by herbivores and fires (which burn faster on lighter vegetation), regularly eliminating seedlings of woody plants. In contrast, woodlands, once established, cannot be easily eradicated by herbivores as woody plants are less palatable. Furthermore, condensation water in the canopy and its accumulation in stems is an effective fire suppressor (Callaway, 1997; Ludwig et al., 1997; Walker, 1993). Like the previous examples, we can identify precipitation and herbivore supression, as an opposite driver to the frequency of heavy fires. Hence, the dynamics of mutual inhibition feedback can be identified in a wide range of ecosystems. Remodeling systems in terms of mutual inhibition allows to apply the concepts of symmetry discussed in this research. Driving such system asymmetric can then help us exploring the roles of single components on the total system. Observations of a shift to an alternative stable state and considerations of system and environmental (a)symmetries provide information about the magnitude of the reverse shift, enabling the prediction of increase and decrease shift magnitudes. However, tipping point locations are harder to predict, such that observation with considering (a)symmetry provides no information about the distance between the tipping points. To our knowledge, this point can only be found by building a symmetric model, leading to decreased biological realism. This characterizes an important challenge, as magnitudes are easier to predict, but tipping point locations are at least as important but may be even harder to predict.

The model of Bush et al. (2017) was created with a specific ecosystem in mind and was strongly biologically justified. To make a symmetric model, we had to change the model of Bush et al. (2017). A functional group (i.e., phototrophic sulfur bacteria) was removed. Moreover, sulfate was not modeled (Bush et al., 2017). Also, trait values for both bacteria are set to the reference values of sulfur‐reducing bacteria, ignoring reference data to cyanobacteria. Furthermore, environmental parameters (i.e., background concentrations and oxidation rate) in Table 1 were tuning parameters to find the symmetric configuration and are thus somewhat artificial. Consequently, the symmetric ecosystem model here shows reduced biological realism. Thus, it will be hard to reproduce a real ecosystem with such symmetric properties and confirm displayed responses experimentally.

Despite not being capable of representing a real ecosystem, our model has potential to be developed further. For instance, our model can include trait variation within functional groups to study symmetry in the biodiversity‐resilience relationship (Limberger et al., 2022). In fact, the model has been implemented with the *microxanox* R‐package (Krug & Petchey, 2023), which has been developed to explore the impacts of varying biodiversity on ecosystem resilience. Thus, comparably little further effort may be required to study symmetry patterns in a more complex system including asymmetry in the amount of trait variation within the two functional groups. Such asymmetry would be expected to lead to higher stability of the state being dominated by the functional group with greater trait variation (though the opposite is possible, Limberger et al., 2022). It would also be interesting to investigate differences in trait variation caused by differences in the relative evolvability (i.e., the capacity for adaptation) between the functional groups. This system (a)symmetry depends on the relative population sizes and relative generation time. We expect the states dominated by the functional group with greater evolvability to be more stable. Again, stability is assessed by comparison with the symmetric case where extent of relative trait variation and evolvability is equal between the functional groups.

In conclusion, a symmetric ecosystem model displays response symmetry, whereas the stronger system or environmental symmetry, the greater the response asymmetry. Increased tolerance of a particular functional group facilitates its persistence and recovery, which gives confidence of a biologically sound system. Collectively, our results suggest that finding symmetric features in real ecosystems may reveal additional understanding and insights about the role of single components and of their interactions and responses to environmental change.

## AUTHOR CONTRIBUTIONS

**Pascal Bärtschi:** Conceptualization (equal); data curation (lead); formal analysis (lead); investigation (lead); methodology (lead); project administration (equal); software (lead); visualization (lead); writing – original draft (lead); writing – review and editing (equal). **Owen L. Petchey:** Conceptualization (equal); funding acquisition (lead); investigation (supporting); methodology (supporting); project administration (equal); resources (lead); software (supporting); supervision (lead); writing – review and editing (equal).

## CONFLICT OF INTEREST STATEMENT

The authors declare no conflict of interest.

### OPEN RESEARCH BADGES

This article has earned Open Data and Open Materials badges. Data and materials are available at https://github.com/pascalbartschi/tipping‐point‐symmetry and https://github.com/UZH‐PEG/microxanox.

## Data Availability

Material, Methods, and Data are available at: https://github.com/pascalbartschi/tipping‐point‐symmetry.
